# Identification of residues/sequences in the human riboflavin transporter-2 that is important for function and cell biology

**DOI:** 10.1186/s12986-015-0008-3

**Published:** 2015-03-14

**Authors:** Veedamali S Subramanian, Rubina Kapadia, Abhisek Ghosal, Hamid M Said

**Affiliations:** Departments of Medicine, Physiology/Biophysics, University of California, Irvine, CA 92697 USA; Department of Veterans Affairs Medical Center, Long Beach, CA 90822 USA

**Keywords:** Riboflavin, hRFVT-2, Mutations, Transport

## Abstract

**Background:**

Riboflavin (RF) is essential for normal cellular metabolic activities. Human cells obtain RF from their surroundings via a carrier-mediated process that involves RF transporters -1, -2 & -3 (hRFVT -1, -2 & -3; products of *SLC52A1, -A2* and *-A3* genes, respectively). Little is known about the structural features of these transporters that are important for their function/cell biology. Our aim in this study was to address these issues for the hRFVT-2, a transporter linked to the neurodegenerative disorder Brown-Vialetto-Van Laere Syndrome (BVVLS).

**Methods:**

We used comparative protein-structure modelling to predict residues that interact with two amino acids known to be critical for hRFVT-2 function (the clinical mutants L123 and L339), site-directed mutagenesis, and truncation approach in the human-derived brain U87 cell model.

**Results:**

First we showed that the defect in the function of the L123 and L339 hRFVT-2 clinical mutants is related to a reduction in protein stability/translation efficiency and to retention of the protein in the ER. Mutating V120 and L121 (residues predicted to interact with L123) and L342 (a residue predicted to interact with L339) also led to a significant inhibition in hRFVT-2 function (with no change in membrane expression); this inhibition was associated with changes in protein stability/translation efficiency (in the case of V120A and L342A) and an impairment in transport function (in the case of L121). Truncating the N- and C- terminals of hRFVT-2 led to significant inhibition in RF uptake, which was associated with changes in protein stability/translation efficiency (it was also associated with a partial impairment in membrane targeting in the case of the N-terminal truncation).

**Conclusion:**

These investigations report on identification of residues/sequences in the hRFVT-2 protein that is important for its physiological function and cell biology.

## Introduction

The water-soluble vitamin B_2_ (Riboflavin, RF) is an indispensable micronutrient for normal cellular function and growth. In its biologically active forms, flavin adenine dinucleotide (FAD), and flavin mononucleotide (FMN), the vitamin plays key metabolic roles as an intermediate in the transfer of electrons in biological oxidation-reduction reactions. Such reactions are involved in carbohydrate, protein and lipid metabolism as well as in the metabolic conversion of vitamins B_6_ and B_9_ into their biologically active forms [1]. Recent studies have also attributed anti-inflammatory and antioxidant properties to RF [2-4]. RF deficiency and sub-optimal levels have been observed in conditions like diabetes mellitus, inflammatory bowel disease, and chronic alcoholism [5-8]; it may also occur as a result of chronic use of certain psychotropic agents [9]. Deficiency/sub-optimal RF levels also occur in subjects with infantile Brown-Vialetto-Van Laere Syndrome (BVVLS), a rare neurological disorder linked to mutations in hRFVT-2 and hRFVT-3; [10-15]. Supplementation with high-doses of RF brings a significant improvement in the clinical symptoms of patients with this disorder [16,17].

Mammalian cells cannot synthesis RF endogenously, and thus, must obtain this essential micronutrient from their surroundings via transport across cell membranes. This event has been known for quite some time to occur via a specific and carrier-mediated process, but the molecular identity of the system (s) involved has only been recently uncovered. There are three RF transporters that operate in human cells, namely hRFVT-1, -2, and -3 (the products of *SLC52A1, SLC52A2* and *SLC52A3* genes, respectively) [18-21]. These transporters share significant identity at the amino acid level, but are differentially expressed in human tissues; they also show differences in their capacity to transport RF [20,22]. Among these transporters, hRFVT-2 appears to be the predominant transporter expressed in brain tissue and is believed to play a key role in regulating brain RF homeostasis [20]. Recent studies have linked mutations in hRFVT-2 to BVVLS, thus providing some insight into structure-function relationship of the hRFVT-2 protein [11,13-15]. Besides that, there is little known about structural features of the hRFVT-2 protein that are important for its function and cell biology. Our aim in this study was to expand our knowledge in these areas, and we used a comparative protein-structure modelling to predict residues that interact with two amino acids known to be critical for hRFVT-2 function, i.e., L123 and L339 [13], site-directed mutagenesis, and truncation approaches. The human-derived brain U87 cells were used as a model in these investigations since the hRFVT-2 protein is predominantly expressed in the brain [20]. Our results showed that the residues located at positions 120, 121 and 342 of the hRFVT-2 polypeptide as well as its N- and the C- terminals to be important for function and cell biology of the protein.

## Materials and methods

### Materials

Human -derived brain U87 cells were purchased from American Type Culture Collection (ATCC, Manassas, VA). GFP-C3 vector and DsRed-ER (ER-marker) were from Clontech (Palo Alto, CA). ^3^H-RF (specific activity: 21.2 Ci/mmol, radiochemical purity: > 98%) was purchased from Moravek Biochemicals (Brea, CA). DNA oligonucleotide mutant primers were obtained from Sigma Genosys. Molecular biology grade reagent and all other chemicals were obtained from commercial vendors.

## Methods

### Site-directed mutagenesis and generation of the N- and C-terminal truncated hRFVT-2 construct

Studies described in the manuscript was reviewed and approved by our institution Research and Development Committee. Point mutations in the open reading frame (ORF) of hRFVT-2 were introduced using the Quick Change site-directed mutagenesis kit (Stratagene, La Jolla, CA). Overlapping primers containing the mutated nucleotides to the specified mutation sites (Table 1) and GFP-hRFVT-2 (WT) fused plasmid were used as a template in site-directed mutagenesis and PCR conditions were followed as described previously [23]. The N- and C-terminal truncated hRFVT-2 constructs were generated by PCR using the primer combinations as shown in Table 2 and conditions specified previously [23]. The PCR products and the GFP-C3 vector were digested with *HindIII* and *SacII*, and products were gel separated and fused to generate in frame fusion proteins with the GFP fusion to the N-terminus of each constructs. The nucleotide sequences of all PCR-generated mutant and truncated constructs were verified by DNA sequencing (Laragen, Los Angeles, CA).

**Table 1:** **Overlapping primers used for generating the specified mutation sites in hRFVT-2**

**Construct**	**Forward and reverse primers (5’-3’)**
GFP-hRFVT-2[F119A]	TTAGCACTGGCC**GCT**GTGCTGGCACTG; CAGTGCCAGCAC**AGC**GGCCAGTGCTAA
GFP-hRFVT-2[V120A]	GCACTGGCCTTT**GCG**CTGGCACTGGCA; TGCCAGTGCCAG**CGC**AAAGGCCAGTGC
GFP-hRFVT-2[L121A]	CTGGCCTTTGTG**GCG**GCACTGGCATGC; GCATGCCAGTGC**CGC**CACAAAGGCCAG
GFP-hRFVT-2[L123P]	TTTGTGCTGGCA**CCG**GCATGCTGTGCC**;** GGCACAGCATGC**CGG**TGCCAGCACAAA
GFP-hRFVT-2[L339P]	TCCTTGGCAGGG**CCG**GGCGGCCTCTCT**;** AGAGAGGCCGCC**CGG**CCCTGCCAAGGA
GFP-hRFVT-2[L342A]	GGGCTGGGCGGC**GCC**TCTCTGCTGGGC**;** GCCCAGCAGAGA**GGC**GCCGCCCAGCCC
Real-Time PCR primers	
hRFVT-2	CCCTGGTCCAGACCCTA; ACACCCATGGCCAGGA
β-Actin	AGCCAGACCGTCTCCTTGTA; TAGAGAGGGCCCACCACAC

The mutated DNA sequences are in bold face text.

**Table 2:** **Primer pairs used for generating the full-length, N- and C-terminal tail truncated constructs of hRFVT-2 by PCR**

**Construct**	**Forward and reverse primers (5’-3’)**	**Positions (bp)**	**Fragment size (bp)**
GFP-hRFVT-2[1–445]	CCC***AAGCTT***ATGGCAGCACCCACGCCCGCC; TCC**CCGCGG**GGAGTCACAGGGGTCTGCACA	1-1335	1335
GFP-hRFVT-2[1–424]	CCC***AAGCTT***ATGGCAGCACCCACGCCCGCC; TCC**CCGCGG**GAACATAGCAACAGCGCC	1-1272	1272
GFP-hRFVT-2[10–445]	CCC***AAGCTT***ATGGTGCTGACCCACCTGCTG; TCC**CCGCGG**GGAGTCACAGGGGTCTGCACA	28- 1335	1307
GFP-hRFVT-2[10–424]	CCC***AAGCTT***ATGGTGCTGACCCACCTGCTG; TCC**CCGCGG**GAACATAGCAACAGCGCC	28-1272	1244

Combination of primers and primer sequence used to generate each construct are shown. The restriction enzyme sites *Hind III* (boldface italic text) and *Sac II* (boldface text) were added to the hRFVT-2 primer to enable subsequent sub-cloning into green fluorescent protein (GFP-C3) vector.

### Cell culture, transient and stable transfection

U87 cells were maintained in DMEM (Invitrogen, CA) supplemented with 10% FBS, penicillin (100,000 U/l), and streptomycin (10 mg/l). For uptake studies, U87 cells were grown in on 12-well tissue culture plates (Corning, NY). For imaging studies, U87 cells were grown in on sterile glass-bottomed Petri dishes (MatTek, Ashland, MA). At 80-90% confluence cells were transfected with 3 μg of GFP-hRFVT-2 (WT), mutated, and truncated constructs with 3 μl of Lipofectamine 2000 (Invitrogen). After 24–48 hrs transient transfection cells were used for imaging. For stable transfection, U87 cells were selected using G418 (0.5 mg/ml) (Invitrogen) for 4–6 weeks as described before [23,24].

### Uptake assay

U87 cells transiently or stably expressing WT, mutated and truncated constructs were grown in 12 well plates and ^3^H-RF uptake assay was performed in Krebs-Ringer (K-R) buffer at 37°C for 3 min (initial linear period; data not shown) following established procedure [23,24]. ^3^H-RF (14 nM) was added to the K-R buffer at the time of uptake assay, and after 3 min the reaction was terminated by ice-cold K-R buffer. We determined the radioactive content in U87 cells using a Beckman Coulter scintillation counter (Fullerton, CA). Protein content of U87 cells was measured using a Bio-Rad D_C_ Protein Assay kit (Bio-Rad).

### Real-time PCR analysis

One microgram of total RNA isolated from stable WT and mutant constructs expressing U87 cells were treated with DNase I and subjected to RT-PCR using iScript cDNA synthesis kit (Bio-Rad). The mRNA expression level was quantified using a real-time PCR machine with specific primers for hRFVT-2 and β-actin (Table 1). Data were normalized relative to β-actin using a relative relationship method [25].

### Western blot analysis

Wild-type, mutated and truncated constructs expressing whole U87 cell-lysate proteins were separated in NuPAGE 4–12% Bis-Tris gradient minigels (Invitrogen), proteins transferred onto immobilon polyvinylidene difluoride membrane (PVDF) (Fisher Scientific), and analyzed by western blotting. The blots were probed with primary anti-GFP monoclonal antibody (1:1000) (Clontech) and β-actin antibody (1:4000 dilutions) (Santa Cruz, CA)]. After three washes with PBS-Tween 20, the blots were probed with the anti-mouse IRDye-800 and anti-rabbit IRDye-680 (both at 1:30,000 dilution) secondary antibodies. Immunoreactive specific bands were detected using the Odyssey infrared imaging system (LI-COR Bioscience, Lincoln, NE) and their intensity was quantified using LI-COR software.

### Confocal imaging of cells expressing the mutated and truncated hRFVT-2 constructs

U87 cell monolayers were imaged using an inverted Nikon C-1 confocal microscopy after 24-48 hrs of post-transfection of GFP-hRFVT-2 (WT), mutated and truncated constructs. The green fluorescent protein (GFP) was excited with the 488 nm line from an argon ion laser and the red fluorescent protein (DsRed) was excited with the 543 nm line from a HeNe ion laser and emitted fluorescence was monitored at 515 ± 30 nm short pass and 570 ± 50 nm long pass filters, respectively. Images were captured with Nikon C-1 software (Nikon Instruments Inc, NY).

### Comparative protein structure modelling

For comparative modeling, we subjected the hRFVT-2 amino acid sequence to PSIPRED fold recognition program (http://bioinf.cs.ucl.ac.uk/psipred) as described before [26]. Among the several templates, we chose the one that covers almost the full-length of the transporter for the most possible three-dimensional models. We further evaluated the model by comparing the score of the energy value to the experimentally determined energy value of native protein of similar length by PROSA (https://prosa.services.came.sbg.ac.at/prosa.php) [27]. The analysis suggested L-fucose-proton symporter (PDB ID: 3O7Q) from *E.coli* [28] as one of the most probable template, although it shares 11% amino acid sequence identity with hRFVT-2. The three dimensional structure of hRFVT-2 generated was visualized by rasmol (www.rasmol.org).

### Data presentation and statistical analysis

All uptake data are means ± SE of multiple separate uptake determinations and are expressed in fmol/mg protein/3 min. The Student’s t-test was used for statistical analysis; P < 0.05 was considered statistically significant. Kinetic parameters of the saturable component of RF uptake were determined by subtracting the diffusing component [determined from the slope of the line between uptake at high pharmacological concentration of RF (500 μM) and the point of origin] from total uptake. The apparent Michaels-Menten constant (K*m*) and maximal velocity (V *max*) were determined using non-linear regression in Graph Pad Prism software (version 5.03). All western blot analyses, real-time PCR assays, and imaging studies were performed on at least three different occasions with different batches of U87 cells.

## Results

### Generation of a comparative model for hRFVT-2 and prediction of interacting residues

**Figure 1:**
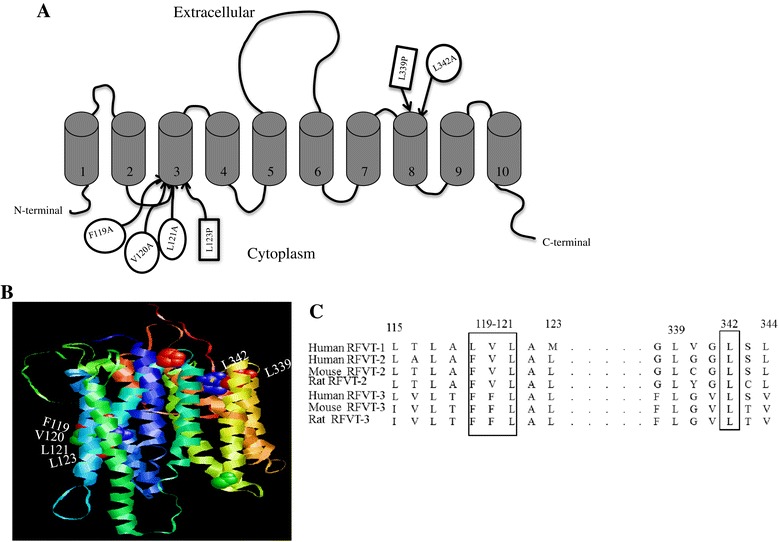
**Predicted membrane topology of hRFVT**-**2 and location of predicted and clinically relevant mutations. A)** hRFVT-2 is predicted to have 10 transmembrane domains with both N- and C-terminal tails oriented intracellularly [20,22]. Predicted and clinically relevant mutations are depicted in oval and rectangular shapes, respectively. **B)** Homology modelling of hRFVT-2 protein to show the location of predicted and clinically relevant mutations in the hRFVT-2 polypeptide. **C)** Comparison of amino acid sequences of human, mouse and rat RF transporters. The locations of the predicted amino acids are shown in box.

The hRFVT-2 is a 445 amino acids protein predicted to have both the N- and C- terminals oriented toward the cell interior [20,22]. Figure 1A shows a schematic representation of the hRFVT-2 predicted secondary structure highlighting the different mutations within its predicted 10 trans-membrane domains [20,22]. From the amino acid sequence, we generated a comparative model for hRFVT-2 as described previously [29]. The topology of hRFVT-2 determined from secondary structure prediction by TMpred program (http://www.ch.embnet.org/software/TMPRED_form.html) matches the relative orientations of amino acids, as determined by comparative three dimensional modelling. According to the *in silico* model, amino acid L123 (which is mutated in BVVLS leading to functional impairment; [13]) is predicted to interact with amino acids F119, V120 and L121 of the hRFVT-2 polypeptide. Similarly, amino acid L339 (another clinical mutant found in BVVLS that led to impairment in function; [13]) is predicted to interact with L342 of hRFVT-2.

### Role of the predicted amino acids in the function of hRFVT-2

**Figure 2:**
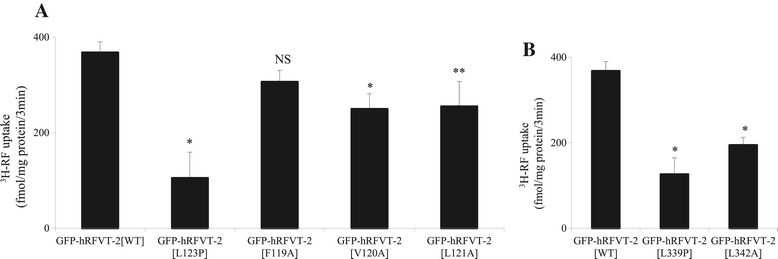
**Effect of hRFVT-2 predicted and clinically relevant mutations on RF uptake by U87 cells. A)** RF uptake by U87 cells stably expressing WT, L123 and its interacting residues (F119, L120 and L121). **B)** RF uptake by U87 cells stably expressing WT, L339 and its interacting residue (L342). ^3^H-RF (14 nM) uptake was performed in K-R buffer (pH 7.4) at 37°C for 3 min. Data are means ± SE of at least 4 independent experiments. *P < 0.01, **P < 0.05.

We examined the effect of the experimental mutants F119, V120 and L121, which are predicted to interact with the clinical mutant L123, on functionality of the hRFVT-2. For that we stably expressed these mutants in human U87 cells, then examined the initial rate of carrier-mediated ^3^H-RF (14 nM) uptake. As seen before [13], the L123 clinical mutant caused a significant (P < 0.01) inhibition in RF uptake. In addition, a significant inhibition in RF uptake was observed in cells expressing the mutants V120A and L121A (P < 0.01 and P < 0.05, respectively) but not those expressing the F119A mutant (for the latter reason the F119A mutant was not considered for further investigations) (Figure 2A). We also determined the effect of mutating the V120 residue of hRFVT2 on kinetic parameters of the RF uptake process. This was done by examining the initial rate of RF uptake as a function of concentration in wild-type and V120 mutant expressing U87 cells. The results showed a marked decrease in the V*max* of the RF uptake process (93.75 ± 8.19 and 51.80 ± 7.89 pmol/mg protein/3 min for wild-type and V120 mutant, respectively) with no change in the apparent K*m* (3.49 ± 0.57 and 2.98 ± 0.86 μM for wild-type and V120 mutant, respectively). With regards to the clinical mutant L339 and its interacting amino acid L342A, both were found to cause a significant (P < 0.01) inhibition in RF uptake by U87 cells (Figure 2B).

### Effect of mutating the predicted (V120, L121 and L342) and the clinically relevant (L123 and L339) residues on level of expression of the hRFVT-2 mRNA and protein

**Figure 3:**
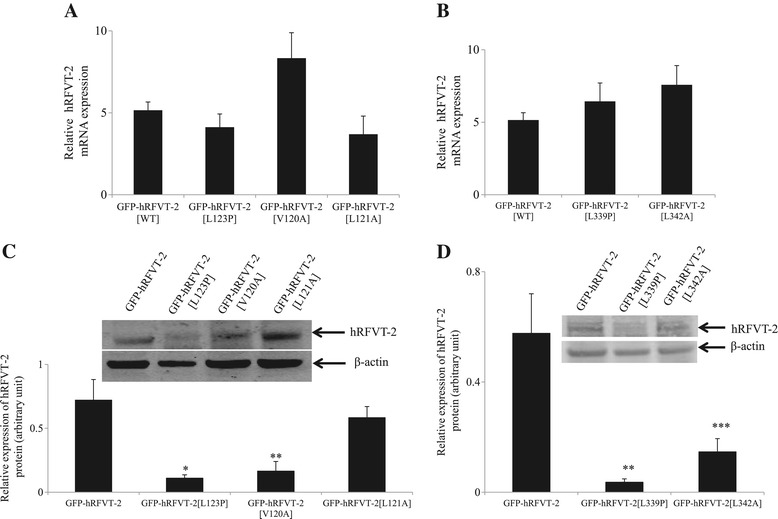
**Effect of hRFVT-2 predicted and clinically relevant mutations on level of expression of hRFVT-2 mRNA and protein in U87 cells. A&B)** Total RNA was isolated from WT, predicted and the clinically relevant indicated mutant constructs stably expressing U87 cells and quantitative real-time PCR was performed. Data (means ± SE) are from at least 3 independent experiments, normalized relative to β-actin. **C&D)** Western blot was performed using equal amounts (60 μg) of total protein isolated from the wild-type, predicted and clinically relevant indicated mutant constructs expressing cells cell-lysates. The blot was probed with the anti-GFP monoclonal and β-actin polyclonal primary antibodies. Data (means ± SE) from at least 3 independent sets of experiment are expressed in arbitrary units. *Inset* is the image of a representative gel. *P < 0.01, **P < 0.02, ***P < 0.05.

To determine whether the reduced levels of ^3^H-RF uptake observed with the different mutants is due to a decrease in transcription, translation efficiency and/or protein stability, we examined the mRNA and protein levels of these mutants following expression in U87 cells; this was done by mean of quantitative PCR and western blot analysis, respectively. Results of the RT-PCR studies showed no significant changes in the level of mRNA expression of the different mutants when compared to level of expression of the hRFVT-2 (WT) (Figure 3A & B). Results of the western blot analysis, which were done using whole U87 cell homogenates, showed that level of protein expression of the clinical mutants L123P and L339P in the total cell homogenates to be significantly (P < 0.01) lower than that of hRFVT-2 (WT) (Figure 3C & D). Similarly, mutating amino acid V120 (predicted to interact with the clinical mutant L123) and amino acid L342 (predicted to interact with clinical mutant L339) were found to lead to a significant (P < 0.02 and P < 0.05, respectively) decrease in level of protein expression in whole cell homogenates compared to hRFVT-2 (WT) (Figure 3C & D, respectively). On the other hand, level of protein expression of the amino acid L121 mutant (which also interacts with the clinical mutant L123) was not affected.

### Live cell confocal imaging of the predicted (V120, L121 and L342) and the clinically relevant (L123 and L339) hRFVT-2 mutants in U87 cells

**Figure 4:**
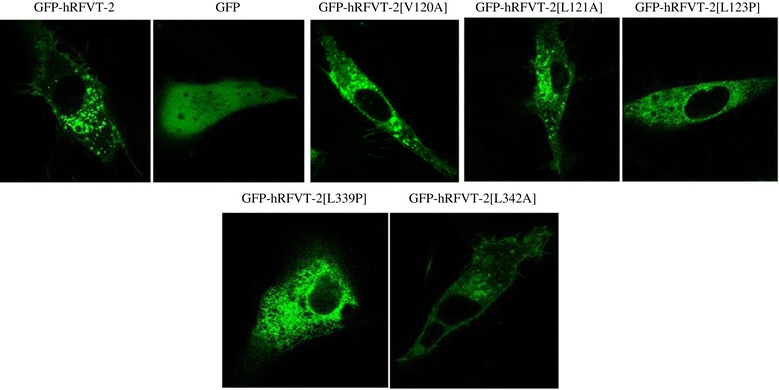
**Cellular distribution of the wild-type, predicted and clinically relevant hRFVT-2 mutants in U87 cells.** Lateral (*xy*) sections of U87 cells stabley expressing WT, predicted and the clinically relevant hRFVT-2 mutants.

**Figure 5:**
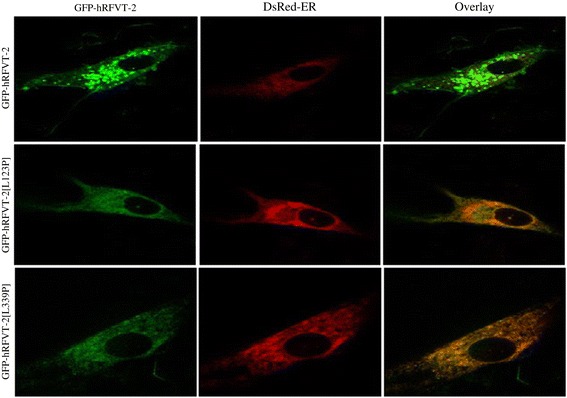
**Co-expression of hRFVT-2 clinically relevant mutants and DsRed-ER in U87 cells.** Co-expression of GFP-hRFVT-2 WT and mutants (left) along with DsRed-ER (middle), displayed overlaid images (right). The co-transfected U87 cells were imaged after 24 – 48 hrs of post-transfection.

To determine the cellular expression of the experimental and clinical hRFVT-2 mutants, we stably expressed the mutant constructs in U87 cells, and performed live cell confocal imaging. The results showed that the GFP-hRFVT-2 (WT) protein is expressed at the cell membrane and is also retained in intracellular vesicular structures, as seen by us previously with other cell-types [30]. Similarly, the experimental mutants GFP-hRFVT-2[V120A], GFP-hRFVT-2[L121A] and GFP-hRFVT-2[L342A] were found to be expressed at the cell membrane and in intracellular vesicular structures in U87 cells (Figure 4). In contrast to the experimental mutants, we found that the clinical mutants GFP-hRFVT-2[L123P] and GFP-hRFVT-2[L339P] were retained intra-cellularlly and they failed to reach the cell membrane (Figure 4). To further validate the cellular localization of GFP-hRFVT-2[L123P] and GFP-hRFVT-2[L339P] clinical mutants, we co-transfected them with an endoplasmic reticulum (ER) marker (ER-targeted red fluorescent protein construct, DsRed-ER) and observed strong co-localization with both GFP-hRFVT-2 clinical mutants and DsRed-ER (Figure 5).

### Role of the N- and C- terminals of hRFVT-2 in function and cell biology

**Figure 6:**
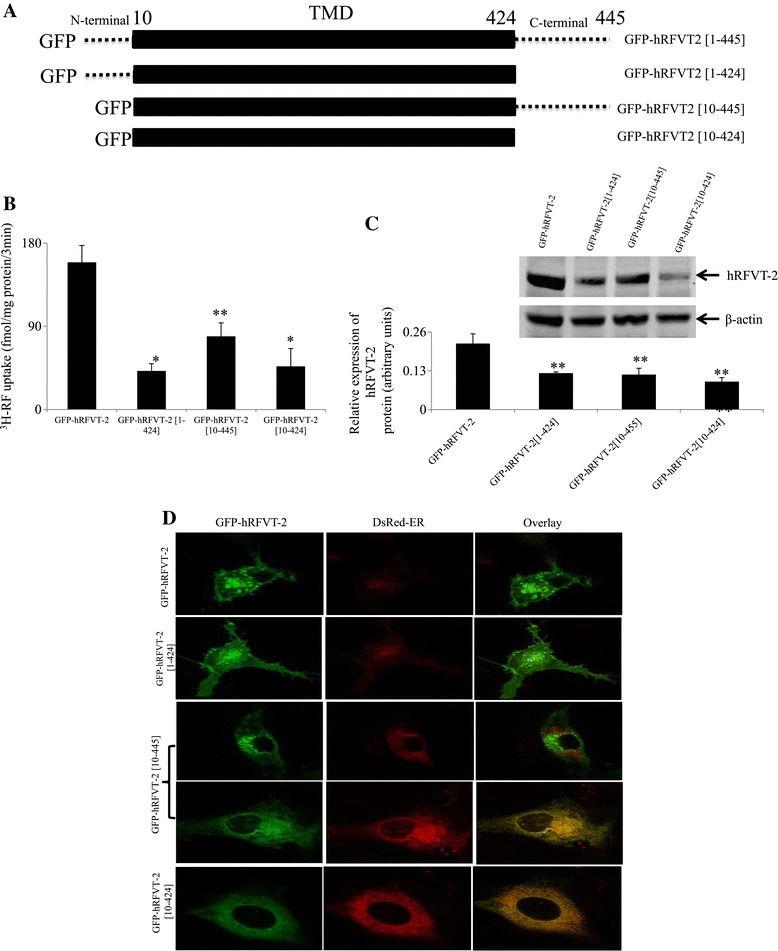
**Effect of hRFVT-2 N- and C- terminal tail truncated constructs on RF uptake and on cellular distribution in U87 cells. A)** Schematic representation of the full-length hRFVT-2, and the N- and the C-terminal truncated constructs with GFP fused to the N- terminus (GFP-hRFVT-2). **B)** ^3^H-RF uptake (14 nM) assay in a WT and truncated constructs transiently expressing U87 cells. Data represents the means ± SE of at least 3 independent experiments. *P < 0.01, **P < 0.02. **C)** Western blot was performed using 60 μg of total protein isolated from the wild-type, and truncated constructs expressing U87 cell-lysates. The blot was probed with the anti-GFP monoclonal and β-actin polyclonal primary antibodies. Data (means ± SE) from 3 independent sets of experiment are expressed in arbitrary units. *Inset* is the image of a representative gel. **P < 0.05. **D)** Co-expressing GFP-hRFVT-2 (WT) (left) along with DsRed-ER (middle), shown as overlaid image (right). Images were obtained after 24–48 hrs of post-transfection.

We examined the role of the N- and C- terminals in the function and cell biology of hRFVT-2. This was done by truncating these sequences (see Figure 6A, for truncation sites) and examining the effect of such truncations on carrier-mediated RF uptake, level of hRFVT-2 protein expression (western blot using whole cell homogenates), and cellular expression of the different hRFVT-2 truncated constructs (live cell confocal imaging) in U87 cells. The results of the functional assay showed that truncating the N- and the C- terminals individually or together led to a significant (P < 0.02 and P < 0.01, respectively) inhibition in the initial rate of carrier-mediated RF uptake by U87 cells (Figure 6B). All three truncated constructs also showed a significant (P < 0.05 for all) decrease in hRFVT-2 protein expression (Figure 6C). The results of the confocal imaging studies showed that a complete truncation of C-terminal tail of hRFVT-2 (GFP-hRFVT-2[1–424]) had no effect on cell surface expression (Figure 6D). On the other hand, the complete removal of N- terminal tail of hRFVT-2 (GFP-hRFVT-2[10–445]) led to a mixed phenotype, with some cells showing expression at the cell surface while others retained the protein in the ER (the latter was confirmed by the significant overlap with DsRed-ER; Figure 6D). When the C-terminal was truncated in addition to the N- terminal (i. e., GFP-hRFVT-2[10–424]), the protein showed complete retention in the ER (Figure 6D). Collectively, these results show that the N- and C- terminal tails are important for hRFVT-2 function and cell biology.

## Discussion

There is little known about structural features of the hRFVT-2 (a transporter whose mutation is linked to BVVLS; [11,13-15]) that is important for its function and cell biology. Our aim in this study was to address these issues and for that we used a comparative protein-structure modelling to predict residues that interact with two residues that are mutated in patients with BVVLS (L123 and L339) and are functionally impaired [13]. We also used a truncation approach to examine the role of the N- and C-terminals in function and cell biology of hRFVT-2. We used the human brain U87 cells as a model since hRFVT-2 is predominantly expressed in brain tissues and is believed to play a key role in regulating brain RF homeostasis [20].

Our comparative protein-structure modelling predicted that amino acids F119, V120 and L121 (all are within TM3) interacts with L123, and amino acid L342 (in TM8) interacts with the L339 of the hRFVT-2 polypeptide. Of those predicted amino acids, L121 and L342 are conserved in all three known human, mouse and rat RF transporters (Figure 1C). We tested the role of all the predicted residues in hRFVT-2 function by examining the effect of mutating these sites on RF uptake. The results showed that mutating V120, L121 and L342 (but not F119) led to a significant inhibition in RF uptake, suggesting that these residues play a role in hRFVT-2 function. The inhibition in RF uptake upon mutating V120, L121 and L342 was not due to an effect on cell membrane expression of the hRFVT-2 (determined by live cell confocal imaging), but rather appears to be due to changes in protein stability/translation efficiency in the case of the V120A and L342A mutants and due to a defect in the transport function in the case of the L121 hRFVT-2 mutant. The kinetic study for the V120 mutant showed a marked decrease in the V*max* of RF uptake by the carrier-mediated process (with no change in apparent K*m*). These results suggest that the V120 mutation affected the number/activity of the hRFVT-2 protein with no effect on its affinity. Our results also showed for the first time that the cause of the defect in the transport function of the L123 hRFVT-2 mutant is due to both a reduction in protein stability/translational efficiency and impairment in cell membrane expression of the protein as indicated by the results of the western blots and the live cell confocal imaging. Similarly, the L339 hRFVT-2 mutant showed a reduction in the level of hRFVT-2 protein expression in total cell homogenate, and also a defect in cell membrane expression, suggesting changes in protein stability/translation efficiency and impairment in membrane targeting. The lack of expression of the L339 hRFVT-2 mutant at the cell membrane is similar to what others have reported previously in renal epithelial cells [15].

Many nutrient/substrate transporters contain sequences/regions that are important for their function and targeting to cell membrane that are embedded within their N-terminal [24,31], C-terminal [23,24,32,33], and/or transmembrane backbone [34,35]. In this study, we show that both the N- (amino acids 1–9) and the C- terminal (amino acids 425–445) sequences of the hRFVT-2 protein are important for its function and cell biology. This conclusion is based on the following experimental observations. First, the complete deletion of the C-terminal region of hRFVT-2 led to impairment in transport function which was, at least in part, due to the observed inhibition in protein stability/translation efficiency; deletion of the C-terminal, however, did not affect plasma membrane expression of the protein. Similarly, removal of the N-terminal sequence of hRFVT-2 led to a significant impairment in RF uptake, which again was associated with changes in protein stability/translation efficiency; it was also, however, associated with a partial impairment in membrane targeting. In contrast, removal of both the N- and the C- terminal sequences of hRFVT-2 lead to a significant inhibition in RF uptake, a reduction in protein stability, and inhibition in membrane expression due to retention of the protein in the ER. Further studies are needed to determine the specific molecular determinants/motifs embedded within the N- and the C- terminals that contribute to normal hRFVT-2 function and cell biology.

## Conclusion

Results of these studies provide new insight into the structural features of the hRFVT-2 protein that play a role in its function and cell biology, and demonstrate an important role for both the N- and C-terminal sequences in this regard.

## Abbreviations

hRFVT: Human riboflavin transporter
BVVLS: Brown-Vialetto-Van Laere Syndrome
PVDF: Polyvinylidene difluoride membrane
GFP: Green fluorescent protein
DsRed: Red fluorescent protein
ER: Endoplasmic reticulum
RF: Riboflavin
